# Phosphodiesterase Inhibitors in Fetal Growth Restriction: Do Not Forget to Consider Fetal Sex and Subcellular Compartmentation

**DOI:** 10.3390/biomedicines12102329

**Published:** 2024-10-14

**Authors:** Anne-Christine Peyter, David Baud, Jean-François Tolsa

**Affiliations:** 1 Neonatal Research Laboratory, Department Woman-Mother-Child, Lausanne University Hospital and University of Lausanne, 1011 Lausanne, Switzerland; 2 Obstetric Service, Department Woman-Mother-Child, Lausanne University Hospital and University of Lausanne, 1011 Lausanne, Switzerland; david.baud@chuv.ch; 3 Clinic of Neonatology, Department Woman-Mother-Child, Lausanne University Hospital and University of Lausanne, 1011 Lausanne, Switzerland; jean-francois.tolsa@chuv.ch

**Keywords:** fetal growth restriction, human umbilical vessels, phosphodiesterase inhibitor, biological sex

## Abstract

Fetal growth restriction (FGR) is a common complication of pregnancy, associated with major perinatal mortality and morbidity, and with an increased risk to develop cardiometabolic diseases later in life. There is currently no effective approach to prevent or treat FGR, despite numerous animal and human studies assessing substances likely to improve fetal growth. Phosphodiesterase (PDE) inhibitors appeared as promising drugs to improve FGR management. However, to date, studies have led to somewhat disappointing or controversial results. In this Opinion article, we would like to draw attention to the need to consider the biological sex and the relative reactivity of human umbilical vein and arteries when developing therapeutic interventions to improve human umbilical circulation using PDE inhibitors. Indeed, we suspect that fetal sex, vessel type and the presence of FGR may influence subcellular compartmentation, which could jeopardize beneficial effects of PDE inhibitors.

## 1. Introduction

It is often said that we are as old as our arteries. But what if we had the “sex” of our blood vessels?

Gender issues are currently in the spotlight, as a means of improving mental health. But individual well-being also depends on physical health, in which the biological sex could play a crucial role, regardless of gender. Sex is an important biological factor, often neglected in clinical and basic research. Indeed, studies using cell culture almost never take into account the sex of the donor [1]. Moreover, most animal studies have been and, unfortunately, continue to be carried out mainly on males only. Similarly, many human studies focus on males, or do not take into account the sex of patients or simply mention the ratio between males and females, but do not analyze the data for each sex separately. As a result, most treatments have been developed based on data obtained only in males. However, there is growing evidence that males and females display important differences in physiological responses, but also in susceptibility to disease, symptoms and even treatment efficacy [2]. This is particularly true in the field of cardiovascular diseases.

Already early in life, human male and female fetuses show differences in the regulation of their umbilical circulation, in both physiological and pathological conditions [3,4,5,6,7]. Namely, fetal growth restriction (FGR) differentially affects male and female umbilical circulation [8,9]. Interestingly, the rate of FGR is higher in female than in male fetuses [10].

### 1.1. Fetal Growth Restriction

FGR is a common complication of pregnancy, associated with major perinatal mortality and morbidity, and with an increased risk to develop cardiovascular and metabolic diseases in childhood and adulthood [11,12]. Growth-restricted fetuses are at increased risk of stillbirth and perinatal complications such as fetal distress or asphyxia. FGR is also associated with a higher incidence of cardiovascular and metabolic diseases later in life, thus contributing to the developmental origins of health and diseases (DOHaD) [13]. This pathology is therefore a public health concern, linked to high healthcare costs worldwide. The mechanisms implicated in the development of FGR remain poorly understood, even though some maternal risk factors have been identified, like maternal diseases (e.g., systemic arterial hypertension or renal insufficiency), malnutrition, maternal stress, strenuous work, as well as tobacco, alcohol and drug abuse. Despite numerous animal and human studies assessing substances that have the potential to improve fetal growth [14,15], there is currently no effective means to prevent or treat FGR and limit its short- and long-term adverse consequences, but only preventive approaches to reduce risk factors, mainly by modifying maternal health behavior [16]. Clinical management of FGR is mainly based on careful monitoring of fetal growth, biophysical profile and doppler velocimetry [12,17,18]. Premature delivery is often the only issue when fetal adaptation is overwhelmed, contributing to further increased risk of perinatal mortality and morbidity. However, there is no systematic screening with a longitudinal fetal growth follow-up in all pregnancies, mainly because of financial considerations. Therefore, identifying high-risk pregnancies remains a challenge [17].

### 1.2. Fetal Growth Restriction and Phosphodiesterase Inhibitors

Phosphodiesterase (PDE) inhibitors, in particular PDE5 inhibitors, appeared as promising drugs in the management of FGR. Many studies investigated their potential to improve fetal growth and reduce the associated adverse perinatal outcomes in humans, whose main findings have been summarized and compared in several recent reviews [19,20,21,22,23]. To date, the most investigated PDE5 inhibitor is sildenafil. Many animal and human studies using sildenafil were performed but led to some conflicting conclusions [22,24,25,26,27].

A recent review of trials using sildenafil in several pregnancy complications, such as maternal pulmonary hypertension, preeclampsia, preterm labor, FGR, oligohydramnios, fetal distress and congenital diaphragmatic hernia, concluded that fetal tolerance and safety outcomes were dependent on the underlying pathology [25]; mild maternal side effects, independent of the clinical indication, were reported. Finally, for most pathological indications, the rationale for prenatal administration of sildenafil was based mainly on limited data obtained in vitro or in rodent animal models. For FGR, some conflicting observations resulted from experimental sildenafil treatment in mouse, rat, rabbit and lamb models, and from several clinical studies [24,25].

An international randomized placebo-controlled clinical trial, the STRIDER trial, used sildenafil in pregnancies with severe early-onset FGR [28]. It was, however, suddenly interrupted due to lack of benefit and unexpected post-natal deaths in the treated group from the Netherlands [29,30]. Indeed, it failed to show any beneficial effect of the maternal sildenafil treatment on fetal growth velocity or birthweight, pregnancy duration, perinatal mortality, or major neonatal morbidity [25,29,31,32,33]. Moreover, the Dutch STRIDER trial reported an increased rate of persistent pulmonary hypertension of the newborn (PPHN) in live born infants in the sildenafil-treated group (18.8%) versus the placebo group (5.1%) [29]. This was surprising as sildenafil can be used in the treatment of PPHN [34]. Although the pathophysiological mechanism associated with the observed increased PPHN rate after prolonged maternal treatment remains unclear, the authors hypothesized post hoc that it could result from a “rebound” vasoconstriction following discontinuation of the treatment [35]. Some suggested that the lack of benefit was due to the administration of an insufficient dose of sildenafil [31,36].

Long-term neurodevelopmental and cardiometabolic outcomes in offspring enrolled in the STRIDER study are still under investigation [37].

## 2. Ex Vivo Assessment of Human Umbilical Vasoreactivity in Appropriate or Growth-Restricted Pregnancies

For many years, we have been interested in the alterations associated with FGR in human umbilical vessels, to better understand the regulation of human umbilical circulation in physiological and pathological conditions, with a particular attention to the influence of the sex of the newborn.

The umbilical circulation is regulated by numerous vasoactive factors, in particular by the nitric oxide (NO)/cyclic guanosine monophosphate (cGMP) signaling pathway [38], which plays a crucial role in the cardiovascular system. NO is a gaseous molecule endogenously produced in the endothelium from L-arginine by the endothelial NO synthase (eNOS). It diffuses into the smooth muscle, where it stimulates the soluble guanylyl cyclase (sGC), leading to synthesis of cGMP, which in turn activates, among others, the cGMP-dependent protein kinase (PKG) to induce vasorelaxation. Intracellular levels of cGMP are tightly controlled by cyclic nucleotide PDEs [39].

Using ex vivo assessment of the reactivity of human umbilical vein (HUV) and arteries (HUAs) in organ bath, we demonstrated that fetal sex is a key determinant in the impact of FGR and PDE inhibition on HUV and HUA vasoreactivity [8,9]. Indeed, FGR is associated with sex-specific alterations in the NO/cGMP-mediated relaxing pathway in the HUV [8]; NO-induced relaxation was impaired in HUV of growth-restricted females compared to appropriate for gestational age (AGA) newborns, whereas no significant difference was found between AGA and growth-restricted males. In contrast, NO-induced relaxation was not affected in HUAs of growth-restricted newborns [9]. Furthermore, although less muscularized, HUV showed a greater reactivity than HUAs to all pharmacological agents we applied. This is interesting as, in the umbilical and pulmonary circulation, veins play a crucial role in supplying oxygen to the body. In particular, HUV carries the oxygen- and nutrient-rich blood from the placenta to the fetus, thus contributing to fetal development.

Moreover, we showed beneficial effects of the non-specific PDE inhibitor 3-isobutyl-1-methylxanthine (IBMX) on NO-induced relaxation in HUV [8].

We therefore investigated the effects of PDE inhibition in HUAs to verify that such a promising intervention to improve blood flow in HUV would not have adverse effects on HUAs.

We found that the effects of PDE inhibition by IBMX vary depending on the vessel type, the sex of the newborn, the presence of FGR, and the vasoconstrictors used to precontract the vascular rings. Direct comparison between HUV and HUAs from each patient showed that, overall, IBMX enhanced NO-induced relaxation in a greater extent in HUV than HUAs [9]. We therefore suspect that, in vivo, the potential resulting imbalance between umbilical venous and arterial blood flow could have adverse effects on fetal hemodynamics. Such observations could be of particular interest for the development of therapeutic interventions using PDE inhibitors.

Surprisingly, most IBMX-sensitive PDE isoforms investigated were more abundant in HUAs than HUV in all study groups [9]. Therefore, as IBMX improved NO-induced relaxation in a greater extent in HUV than in HUAs, despite a higher IBMX-sensitive PDEs protein content in HUAs, we hypothesized that subcellular compartmentation could play an important role in the regulation of human umbilical vascular tone. Indeed, subcellular compartmentation appears to be particularly important in cyclic nucleotide signaling in vascular smooth muscle cells [40], and cGMP compartmentation was previously described in HUA smooth muscle cells [41]. Our data suggest that this subcellular organization might differ between HUV and HUAs, but also depend on the fetal sex and the presence of FGR. However, to date, there are no studies comparing subcellular compartmentation between HUV and HUA, nor the influence of fetal sex or the presence of FGR.

## 3. How Could Ex Vivo Data Provide Clinicians with New Insights?

Extrapolations of our observations in the in vivo situation must, of course, be made with caution. Indeed, our study was based on the use of a non-specific PDE inhibitor, whereas in vivo more specific agents are usually administered to limit side effects. In addition, it is not known how much of the drug can cross the placental barrier. In any case, it seems likely that the drug concentration will be lower in HUAs than in HUV, further reducing its effect, which seems already attenuated in HUAs.

Although based only on term newborns and on the use of IBMX rather than sildenafil, our findings may provide some insights into the lack of benefit observed in the STRIDER trial.

The increased rate of PPHN observed in the Dutch STRIDER was attributed, post hoc, to a potential “rebound” vasoconstriction following cessation of the treatment [35].

Based on our data [9], another potential explanation could be that sildenafil treatment could lead to some imbalance in the umbilical circulation by promoting relaxation in HUV more than in HUAs, thus resulting in unexpected adverse effects on the fetal hemodynamics. To verify our hypothesis, it would be of course necessary to investigate the effects of sildenafil on HUV and HUA vasoreactivity. Interestingly, the UK STRIDER reported a greater proportion of deteriorated ductus venosus blood flow in the sildenafil-treated group [31], suggesting a deleterious effect on fetal hemodynamics. Measurement of sildenafil concentration in HUV and HUAs just after birth in the STRIDER trial would have been useful to know the respective concentrations achieved in vivo with the maternal treatment. Moreover, Doppler measurements in both HUV and HUAs in pregnant women treated with sildenafil would also have been of interest to document the effects of this drug on the umbilical circulation in vivo, because most clinical studies unfortunately limited those investigations to HUAs. To our knowledge, no study has directly compared the effects of sildenafil on HUV and HUA in vivo.

These observations could be useful for further reflection about PDE inhibitors’ administration; although these drugs are widely used to treat several pathologies without major side effects, any new indication should be preceded by extensive in vitro/ex vivo investigations to evaluate potential beneficial and side effects on the whole targeted system. Both physiological and pathological conditions should be considered, as well as the sex of the patient. Moreover, it would be necessary to ensure not only that the targeted PDE isoform is present, but also that it is able to interact in the native tissue with the targeted signaling pathway. Indeed, if there is an uncoupling between the targeted PDE and the cGMP and/or cAMP signaling pathway, due to subcellular compartmentation, administration of the inhibitor would likely have no or little beneficial effect.

As underlined by Smith in his comment about the STRIDER study, a better knowledge of the mechanisms contributing to FGR and the influence of the fetal sex would help to identify strong candidates for interventional studies [42].

To our knowledge, there are no other studies directly comparing the effects of PDE inhibition on NO-induced relaxation between HUV and HUA, nor the influence of the fetal sex or the presence of FGR, which could support our hypotheses.

Nevertheless, as we found that the effects of PDE inhibition vary according to the sex of the fetus, studies that group males and females may miss significant differences. It is therefore likely that a re-analysis of data from previous studies that did not differentiate males and females could highlight some interesting findings.

## 4. Conclusions

In conclusion, the effects of PDE inhibition vary depending on the sex of the newborn, the presence of FGR, the vessel type and vasoconstrictors acting on the vessels. This finding draws attention to the need for caution in the development of therapeutic interventions based on the use of PDE inhibitors to improve the placental-fetal circulation. In particular, fetal sex and both umbilical vein and arteries should be considered when designing therapeutic interventions to improve the human umbilical circulation.

Further investigations on the contribution of subcellular compartmentation to the regulation of the human umbilical vascular tone may provide a better understanding of how to improve fetoplacental perfusion while maintaining a balance between blood flow in the HUV and HUAs. This may allow for the design of effective therapeutic strategies to prevent or limit the development of FGR and its short- and long-term consequences.

More broadly, these findings add to the growing evidence supporting the need to consider biological sex—in most cases sex assigned at birth—as an important biological variable in cardiovascular research. With a view to personalized medicine, future research should also investigate the extent to which the influence of this parameter could be modulated, for example, by possible hormonal variations, natural or interventional, linked to gender variations. However, this could be relevant in cardiovascular medicine but would have only limited application in the field of perinatal research, with the exception of intersex individuals.
